# On the Use of the Essential Oil of Lemons in Various Inflammations of the Eye; with Cases

**Published:** 1833-07

**Authors:** John Foote


TRANSACTIONS OF THE MEDICO-BOTANICAL SOCIETY OF LONDON.

*Published under the Direction of the President and Council.*

### ESSENTIAL OIL OF LEMONS.

### *On the Use of the Essential Oil of Lemons in various Inflammations of the Eye; with Cases.* By JOHN FOOTE, jun. Esq., L.S.A., A.M.B.S.

(Read June 11th, 1833.)

FOR several years past the use of stimulants in various external inflammations of the eye has been gradually gaining ground. An opinion formerly prevailed, not only among the public generally, but also very extensively in the profession, that the eye was a very tender organ, and would not bear rough handling. This idea tended in a great measure to retard the period when stimulant applications were first employed, and to induce great caution in their use. It was formerly the practice, in many inflammatory affections of this organ, to bleed, cup, and leech, to such an amount as frequently even to do serious injury to the constitution of the patient; such mischief indeed, that he would be years in getting over it, and perhaps might never entirely recover from its effects. Even lately, within a few years, a work has been published by Mr. Lawrence, one of the surgeons to St. Bartholomew's Hospital, on the Venereal Affections of the Eye, recommending, in the gonorrhœal ophthalmia, that blood should be drawn as long as any could be obtained from a vein! And what does he present us with as the results of his depleting practice? Truly, a melancholy list of lost eyes. On the other hand, if we look to the reports of Mr. Guthrie on the stimulant plan of treatment in the same affection, we find success attend his practice. I have seen about six or seven cases of this highly dangerous ophthalmia; they have been all treated on the stimulating plan, and have all invariably ended successfully. Having been a pupil at the Royal Westminster Ophthalmic Hospital for a period of nearly five years, I have had ample opportunities of testing the relative value of the stimulating and depletory plans of treatment, and, were all things equal, the fact that the former saves the patient from that abstraction of blood which is frequently urged to a great amount, would alone be sufficient with me to give it the preference: but it has other advantages; it effects a cure in a shorter period, and does not leave behind it, as the antiphlogistic plan generally does, a low or chronic inflammation of the parts, requiring a stimulant application to remove it.

To Mr. Guthrie belongs the credit of having introduced, and followed up to a very great extent, the plan of stimulation in acute external inflammations of the eye. Before his time, the vinum opii, solutions of nitrate of silver in distilled or rose water of various strengths, the unguentum zinci, or the unguentum hydrargyri nitratis dilutum, were employed, but not extensively, in the chronic inflammations; while Mr. Guthrie has used them, and stronger stimuli, in the acute stage of the same affections; a step, for the boldness of which he would have been severely blamed, but for the success with which his efforts were crowned. On the continent, Graafe and Walther, and some other surgeons, employed the solution of the nitrate of silver in purulent ophthalmia; but this application has the inconvenience, that, when employed for any length of time, it has the effect of staining the eye. This was first pointed out in print by Dr. Jacob, in the Dublin Medical Transactions, but was constantly inculcated at the Ophthalmic Hospital in Warwick street, to my certain knowledge, both by Dr. Forbes and Mr. Guthrie, the physician and surgeon to that institution.

In the May of 1827, Mr. Guthrie pushed his inquiries still further: he caused an ointment to be made with ten grains of the nitrate of silver to the drachm of simple ointment, and tried this in numerous cases. After several formulæ, variously modified, had been essayed, he finally decided on a preparation containing the argentum nitratum with Goulard's extract, mixed up with lard; he also employed an ointment made with the oxymuriate (the bi-chloride) of mercury, and another composed of simple cerate and the sulphas cupri, five grains to the drachm; but these latter he soon abandoned. These applications were employed by him in the various ophthalmiæ, acute and chronic, affecting the conjunctiva, the cornea, and the sclerotica; also in cases of nebula; but in these latter affections the milder stimuli are more properly required, as the disease generally takes a long time for its removal, and a milder, and at the same time a more frequently renewed, stimulus has a greater effect in exciting the absorbents to action. The celebrated French surgeon, Dupuytren, employs in this disease a combination of equal parts of calomel, sugar, and oxide of zinc, blown into the eye. Various other surgeons have tested the powers of these applications, and all speak in their favour; some more sparingly than others. Mr. Mackenzie, of Glasgow, is one of those who do not speak so highly of these remedies as they deserve; which need not excite our admiration, as he has hitherto employed a bad preparation. Mr. Lawrence has every wish to give the stimulant treatment a fair trial, but he has not as yet employed it in any case.

The theory on which this peculiar plan was first introduced was founded on an opinion of John Hunter's, which has become almost an axiom in medicine, viz. "that no two diseases can exist at one and the same time in the same constitution." That which he applied to the system generally has been adopted in reference to a single organ of that system: in consequence, it was conceived that, by exciting a more severe inflammation in the eye than that which already existed, but at the same time of shorter duration, a cure would be effected of the original malady. Whether this theory be or be not a valid one, it matters little, so long as the treatment deduced from it be successful; and that it has hitherto been attended with success, is amply proved by the records in the various medical journals of this and other countries, and by the case-books of the various ophthalmic institutions, where it has been fairly tried. In saying this, I am not alluding to any particular formula, to any peculiar preparation, but to all those remedies which come under the general head of external stimulants. Neither do I mean to aver that in no cases has it been unsuccessful: in some few, probably from idiosyncrasy, or extreme nervousness, the stimulant application appears to have done harm, and was necessarily abandoned; but these serve only to form the exception to the rule.

Under such circumstances, my ophthalmic lore having been acquired in a sthenic school, if I may so term it, it will scarcely be wondered at that, knowing from experience the stimulating properties of the essential oil of lemons, when in contact with the conjunctiva, I should be ready to admit its powers in the treatment of ophthalmia, and anxious to test its properties. For this purpose, the Royal Westminster Ophthalmic Hospital offered me an ample field.

In the year 1829, the following paragraph, which appeared in the *Collectanea* department of the London Medical and Physical Journal, under the head "Surgery," first directed my attention to this essential oil as a remedial agent in affections of the eye.

"*Efficacy of Lemon-juice in some Diseases of the Eye.* (From the *Journal für Chirurgie und Augenheilkunde,*) M. Werlitz thus employs this novel remedy. He cuts a slice of lemon-peel about an inch long and half an inch broad, places the outer part opposite the affected eye, the eyelids being opened, and by slight pressure squeezes out the little drops of volatile oil contained in the tissue of the rind into the eye. The sensation produced is acute, and continues for an hour or two. If the pain caused should be severe, cold applications are to be employed. The oil of the lemon-peel appears to increase thecapillary circulation, and to cause the absorption of morbid depositions.

"From experiments which have been made at La Charité at Berlin, it appears that the following diseases are remedied by this treatment: 1. Inflammations of the eye which are passing into a chronic state, and which affect the external parts, as the conjunctiva, cornea, or sclerotic, particularly if the small vessels are turgid. M. W. has also found the remedy useful in rheumatic, gonorrhœal, and scrofulous ophthalmia. 2. In pannus and pterygium. 3. In albugo and opacity of the cornea. 4. When the texture of the cornea has lost its healthy density, and becomes soft and spongy. The remedy may be employed frequently during the day, depending upon the degree of irritation it produces. M. W. relates seven cases of cures of various diseases of the eye effected by this treatment."

There are perhaps few persons who are unacquainted with the stimulant properties of the essential oil contained in the follicles of the bark or rind of the lemon or orange, as, in peeling these fruit, it frequently happens that a follicle or two burst, and a particle of the essence gets lodged in the conjunctiva. It was probably an accident of this kind which first induced Mr. Werlitz to try its powers in inflammations of the eye, and thus add another remedy to the list of the ophthalmic materia medica. Having obtained the permission of Mr. Guthrie at a period far antecedent to the time when I actually availed myself of it, I have recently given this essential oil a trial at the Ophthalmic Hospital, in various inflammations, more especially the catarrhal, which the peculiar constitution of the atmosphere at the present time has rendered epidemic. In the majority of cases in which I applied it, I found it successful; in a few it appeared not to be of use, and the application of cupping-glasses to the temple was ultimately required. It generally caused pain, varying in duration from half an hour to three, and also in intensity in different individuals. In one person, a gentleman of the medical profession, having increased vascularity of the eyelid, and who had been accustomed to have the vinum opii applied, it excited pain to such a degree that he declared his eye felt as if on fire; but this unpleasant symptom went off in about ten minutes; another person, to whose eye I applied it, said that the pain it occasioned was trifling, and not at all to be compared with that which arises from the application of the vinum opii. Such is the difference of sensibility in different individuals; and I may add, that both experienced benefit from it. I have deemed it necessary, in general, to administer aperients and other medicines, in some of the cases in which I employed it, according to the severity of the symptoms indicating general irritation.

M. Werlitz does not apply the oil at all in a scientific or satisfactory manner. His method of squirting the essence from the rind appears to me to be both rude and coarse, and could scarcely be employed in private practice; I have therefore adopted another way, which I shall shortly mention. In strumous ophthalmia, it is exceedingly difficult, from the tumefaction of the eyelids and the restlessness of the child, so to separate the eyelids as to obtain a view of the eye itself, and, under such circumstances, it would be next to impossible to keep them open a sufficient length of time, until the operator shall have properly injected the drop. In the trials to which I have subjected it, I have invariably used the essential oil of lemon of the shops, and have dropped it in the eye in the same manner that the vinum opii is applied, namely, with a quill cut in the shape of a pen, but rounded off, instead of having a point. It requires to be dropped in very speedily, as it rapidly volatilizes. I have always, when it has been in my power, applied the essence once a-day, but it has occasionally happened that the patients have neglected attendance for two, three, or more days, and have then returned much worse than they were previously. I may also add, that occasionally, even when they were regular in their attendance, a relapse would take place; and this occurred in a most remarkable manner, on Saturday the 1st of June. On the preceding day, several cases were reported as rapidly improving, or nearly convalescent, and yet, on the 1st, with perhaps one exception, they returned with a fresh attack. Had only one or two cases relapsed, I might have attributed it to negligence and inattention to my directions on the part of the patient, or to exposure to cold; but, as by far the majority of the cases were similarly affected, I could not avoid considering it as something remarkable, and dependent on some change in the atmosphere.

In summing up, I may say, I have employed the essence of lemons in various acute and chronic inflammations, in opacity of the cornea, and the purulent and muco-purulent ophthalmia, but not in pannus, pterygium, or albugo; cases of the latter description not having come under my care latterly. I should consider, from the nature of the complaints, that it may prove advantageous in albugo, but in regard to the other two diseases, I should be very dubious of its powers. On the whole, I believe it to be preferable to the vinum opii in all cases where a stimulant is indicated, and equal, in many, to the unguentum argent. nitratis, but falling short of it in others; while the great facility with which it may be obtained and applied, renders it a great acquisition to the country surgeon, who would find it inconvenient to spend a couple of hours over the mortar in pulverizing the nitrate of silver, with the prospect that, when he has made his ointment, he may be unable to use it, from the circumstance that he has left a few granules of the nitrate not sufficiently fine, and which no after-treatment will be able properly to reduce. I have tried it in about five and twenty cases; and shall subjoin the detail of a few, with which I shall conclude.

Case I. *Catarrhal Ophthalmia, treated by the Oleum Limonum.*

Sarah Lawrence, ætat. twenty-three, admitted the 17th May, 1833. Catarrhal inflammation of the left eye, of a month's duration. The disease was not noticed for the first week, but was afterwards treated by a surgeon; leeches were applied to the temple, a blister behind the ear, lotions, and medicines internally; she says the eye improved a little under the treatment pursued. She complains of pain in the eye and parts adjacent, so severe as nearly to prevent her sleeping at night; considerable lachrymation, tears scalding; vision misty; the eyelids used to adhere in the morning, but not for the last two or three days. Her general health is somewhat affected by the disease, appetite failing, tongue rather furred, bowels open. The conjunctiva of the ball and eyelids injected and inflamed, the affection of the lids being the more intense; a pustule forming on the lower margin of the cornea. The vessels of the conjunctiva of the ball are more of a pink colour, but do not form the zone around the cornea. Neither the cornea nor the iris are affected.—App. Ol. Ess. Limon. gutt. ad ocul.: ℞. Magnes. Sulphatis ℥i.; Infusi Sennæ comp. lb.ss. solve. Capiat coch. larga ij. omni mane primo.

20th. The pain and inflammation are diminished, and vision is improving; the pustule is small; bowels opened by medicine.—Rep. Ol. Limon. et Mist. purgans.

21st. Inflammation very much diminished.—Rep. Ol. Limon.

22d. The pain has nearly gone; vision considerably improved. —Rep. Ol. Limon. et Mist. purgans.

23d. Vision is as perfect as ever. Slight inflammation of the eyelids still exists.

27th. Is going into the country. To have the vinum opii dropped in occasionally.

Case II. *Catarrhal Inflammation in a Strumous Constitution, treated by the Oleum Limonum and Alteratives.*

William Minnifit, ætat. three, was admitted May the 17th, 1833; a child of strumous constitution, and apparently in ill health. The conjunctiva of the eyelids and ball of the left eye are very much inflamed, the cornea slightly participating, and having a small speck near the centre; there is considerable lachrymation, tears scalding, attended with pain and intolerance of light; he cannot open the eye. The bowels are not regular, and he is rather feverish.—App. Ol. Limon. ℞. Hyd. cum Cretâ, gr. ij.; Pulv. Rhei, gr. ij. M. fiat pulv. sumat i. nocte maneque.

20th. His vision is improved, and he can bear the light better; the pain and inflammation are diminished; bowels open.—Rep. Ol. Limon. et Pulv.

22d. Improving.—Rep.

27th. Has not attended since the 22d. The inflammation is greater, and all the symptoms are materially increased in severity.—Rep. Ol. Limon. et Pulveres, nocte maneque sumend.

31st. Rather better.—Cont.

June 3d. Nearly well.—Rep.

5th. Dismissed, cured.

Case III. *Pustular Inflammation, treated by the Ol. Limon.*

Frederick Plumber, ætat. six, admitted 17th May, 1833; a child of a strumous habit of body. Has pustular inflammation of the right eye, of five days' duration. There is a pustule on the outer, and another on the inner margin of the cornea, attended with inflammation of the conjunctiva of the ball and lids. There is not much pain at present. Has merely applied a bread and water poultice, and taken aperient medicine.—App. Ess. Limon. Pulv. Hyd. cum Cretâ et Rheo i. nocte maneque sumend.

20th. Is nearly well; the pustules are scarcely visible.—Rep.

22d. Dismissed.

Case IV. *Pustular Inflammation, treated by the Ol. Limon.*

Mary Mirvin, æt. six, admitted May 24th, 1833, with pustular inflammation affecting the lower margin of the cornea of the left eye, attended with vascularity of the conjunctiva covering that part; considerable lachrymation and pain. The disease has existed a month; child otherwise healthy.—App. Ol. Limon. Pulv. Hyd. cum Cretâ, cum Rheo, n. m. sumend.

25th. There is less pain, and vision is improved; the pustule is smaller, and the vascularity is diminished.—Ol. Limon. et pulveres.

27th. Improving. Bowels not open.—Rep. Oleum Limon. ℞. Magn. Sulph. ℥i. fiant pulveres tres, sumat i. p. r. n.

June 1st. Nearly well.—Rep.

3d. Rep. Oleum, et Magnesiæ Sulphas.

7th. Dismissed.

Case V. *Opacity of the Cornea, treated by the Ol. Limon.*

Fanny Folkes, ætat. eleven, was admitted an in-patient in the Westminster Hospital, with opacities on each cornea. Her eyes had been bad for the period of two years, the disease being the result of inflammation. Her vision is very imperfect; she cannot distinguish letters. The opacities are nearly central, attended with a general muddiness of the cornea. Has been under treatment for some time, but without deriving any advantage. There is not any inflammation of the eye, or its appendages, at present.

June 7th, (ten days after admission.) The essential oil of lemons has been applied daily, with the effect of producing considerable smarting pain, lasting about a quarter of an hour. The general muddiness of the cornea is much diminished; the nebulæ are not so evident as when she was admitted, and her vision is so much improved that she can distinguish the letters, and spell them, on her admission-ticket. Her bowels have been regulated by medicine occasionally.—Let her continue.

CASE VI. *Muco-purulent Ophthalmia, followed by Erysipelas of the Face, extending to the Eyelids, treated with the Ol. Limon. and the Tartar Emetic.*

Timothy Fitzpatrick, ætat. fifty, a man of a sanguineous temperament, short and stout, has been in attendance a considerable period.

About two years ago, he lost the sight of the left eye from inflammation, in consequence of which the cornea and other parts became disorganized, and the cavity of the anterior chamber lost, the cornea lucida being completely flattened. He has been an out-patient occasionally several times since, for attacks of inflammation affecting the conjunctiva covering the ball and lining the eyelids.

During the night of the 14-15th of May, 1833, he suffered from a fresh accession of pain in the left eye, attended with a considerable discharge, the pain chiefly at the outer canthus. He does not know any cause for this attack; says he has not caught cold lately, nor has anything got into the eye that he is aware of. On examining the organ (May 15th,) there appears considerable inflammation of the conjunctiva of the ball and lids, that membrane being exceedingly vascular and chemosed; a fluid of a muco-purulent character is constantly and rapidly secreted; the pain continuing at the outer canthus. [Several symptoms are here either wanting or masked, in consequence of the loss of vision; such as the aversion to, and the inability to bear, the light, the degree to which vision is affected by the inflammation, &c.] The tongue is covered with a whitish fur, and the bowels are regular.—Applicatur gutta Olei Essentialis Limonis. Capiat Magnesiæ Sulphatis ℥i. mane.

16th. The application of the drop did not cause much pain, nor did it last any length of time. The eye felt easier for some time after, the original pain in the canthus beginning to return about nine in the evening, when he went to bed. He could not sleep, from the pain. The discharge appears to be rather increased, and there is a blush of erysipelatous inflammation on the left cheek, extending to the eyelids, which are tumefied and inflamed externally and internally; the cheek and eyelids are puffy; skin hot; no pain in the head, but it continues at the outer canthus, rather however as soreness than actual pain; the tongue is covered with a dirty-brown fur; a slight acceleration of pulse. The salts operated well.—Rep. Ol. Limon. App. Ung. Cetacei palpebris nocte. Aqua tepida sæpe indies injicienda. ℞. Antimonii Tartarizato, grana duo; Magnesiæ Sulphatis, unciam; Aquæ, uncias octo. Solve, fiat mistura. Capiat cochlearia ampla duo omni horâ.

17th. The drop gave him considerable pain. He passed a better night. There is not so much tumefaction of the lids, nor general inflammation; he thinks that there is more discharge, but that it is thicker. The mixture opened the bowels freely, and excited slight nausea; tongue furred.—Rep. Ol. Limon. Lotio Aluminis sæpe utend. Rep. mixture, sumatur dosis omni bihorâ.

19th. The tumefaction has nearly entirely subsided; he can open the eye very easily; sleeps well; the inflammation of the eye abating, and discharge lessening; the erysipelatous blush has almost gone, as likewise the discoloration of the eyelids; tongue continues furred; bowels open.—Persistet in usu omnium medicamentorum.

22d. Improving.—Rep.

From this date to the 3d of June he gradually improved; there remained then very little discharge, and that depending on a granular state of the eyelids, which has latterly come on. No pain or uneasiness.—Ordered the Sulphas Cupri, to remove the granulated appearance of the lids.

Case VII. *Purulent Ophthalmia, dependent on Leucorrhœa in the Mother; Use of Ol. Limonum.*

David Davis, aged six weeks, was admitted May 22d, 1833, with purulent ophthalmia of both eyes, of five weeks' duration. The mother has had leucorrhœa for some time previous to parturition, and it still continues on her. The child's eyes appeared weak soon after birth, but she could not ascertain the exact period when they became inflamed. Within a week however after birth, purulent matter, of a proper consistence and yellowish colour, was secreted abundantly from each eye, the lids at the same time being very much tumefied. The mother has never done anything for them, save cleansing them now and then with warm water. There is at present considerable discharge of a tenacious, yellowish matter, attended with inflammation of the eyelids, which are not so tumefied as they were; the inflammation extends, but in a milder degree, to the ball of the eyes; on the centre of the corneæ there is a speck already formed. The child can open the eyes, but not easily; its health has suffered very much; the bowels are open, but the stools are of a dark colour, and the little patient is very much emaciated.—Applicetur Oleum Limonum ocul. sing. Pulv. alter. i. nocte maneque sumendus. To continue the injection of warm water between the eyelids, and that frequently.

24th. The discharge is very slight; can open the eyelids easily; the eyeballs appear to be nearly clear from inflammation, which is also lessened on the lids. The bowels are open; stools of a greenish colour. It appears that, by mistake, the wrong powders were given, and the child had a large dose of the tartarized antimony, instead of the alterative powder which had been directed.—Rep. Ol. Limon. Pulv. alterat. ss. n. et m. sumend.*

25th. Is still much affected by the powders, the same kind

* When this order was given, the mistake already recorded was not ascertained to have happened.

having been given as at last report. There is rather more discharge from the left, but very little from the right.—Rep. Ol. Limon. Not to have any powders.

27th. A little better.—Rep. Ol.

28th. Discharge lessened; is altogether improved.—Cont.

31st. Nearly well.—Rep.

June 1st. There appears to be an increase of discharge, and the child is not so well.—Rep.

4th. There is more discharge and inflammation, and the lids are again tumefied.—Rep. Ol. Limon. Hyd. cum Cretâ, gr. ij. Pulv. p. r. n. sumend.

6th. Discharge much diminished.—Rep.

7th. Very little purulent matter secreted.—Cont.

9th. Nearly well.—Rep.

